# Retraction: MicroRNA98 acts as a tumor suppressor in hepatocellular carcinoma via targeting SALL4

**DOI:** 10.18632/oncotarget.28884

**Published:** 2026-05-20

**Authors:** Wuyuan Zhou, Benkui Zou, Lisheng Liu, Kai Cui, Jie Gao, Shuanghu Yuan, Ning Cong

**Affiliations:** ^1^Department of Hepatobillary Surgery, Shandong Cancer Hospital, Jinan, Shandong 250117, P.R. China; ^2^Department of Urology Surgery, Shandong Cancer Hospital, Jinan, Shandong 250117, P.R. China; ^3^Clinical Laboratory, Shandong Cancer Hospital, Jinan, Shandong 250117, P.R. China; ^4^Department of Radiation Oncology, Shandong Cancer Hospital, Jinan, Shandong 250117, P.R. China; ^5^Department of Intervention Therapy, Shandong Cancer Hospital, Jinan, Shandong 250117, P.R. China

This article has been retracted: This decision follows an investigation by Oncotarget Scientific Integrity office, that revealed multiple image duplications across several figures, involving unrelated articles published before, concurrently, or after the submission. Specifically:

- Transwell Assay Images: Images in Figure 3C, 3D were found in reference [1] (Figure 3B and Figure 6B), [2] (Figure 4C), [3] (Figure 6), [4] (Figure 3C, 3D), and [5–6] (Figure 2C and Figure 4C). Additionally, Figure 2D and Figure 4C of [7] and Figure 4B of [8] contain these duplicates. Images from Figure 7C, 7D were also found in a 2015 article [9] and 2016 articles [4–6, 10].
- Wound Healing Assay Images: Images in Figures 3A, 3B, 7A, and 7B were found in a 2015 article [11] (Figure 4), [12] (Figures 4D and 5C), and in later publications.
- Western Blot Images:
- Figure 4A blots match reference [13] (Figure 2B) and [8] (Figure 4I).
- Figure 4B (MMP2) matches a 2013 article [14] (Figure 4B as Bax/CHG), a 2015 article [9] (Figure 5B as Robo1), [15] (Figure 4D), and [8] (Figure 4I).
- Figure 4C, 4D blots are duplicated in [16] (Figure 3E), [8] (Figures 3H and 4A), and [15] (Figure 2C).
- Figure 5F (Src) is a duplicate of a 2013 article [14] (Figure 6).
- Figure 8A–8C were found in a 2016 paper [16] (Figures 1C and 6E).
- Tumor Images: Figure 10C images are identical to a 2015 article [17] (Figure 7C).

As the authors have not responded to previous multiple requests for comment or provided the original data, the journal is proceeding with the retraction. While we have attempted to reach all co-authors regarding this matter, none of the authors could be reached.

Original article: Oncotarget. 2016; 7:74059–74073. 74059-74073. https://doi.org/10.18632/oncotarget.12190
